# Household drug-resistant TB contact tracing in Tajikistan

**DOI:** 10.5588/ijtld.23.0066

**Published:** 2023-10-01

**Authors:** M. L. Rekart, A. Aung, T. Cullip, W. Mulanda, L. Mun, B. Pirmahmadzoda, J. Kliescokova, J. Achar, J. L. Alvarez, N. Sitali, A. Sinha

**Affiliations:** 1 Médecins Sans Frontières (MSF), Dushanbe, Tajikistan; 2 MSF, London, UK; 3 Dushanbe City TB Center, Dushanbe, Tajikistan; 4 Department of Global Public Health, Karolinska Institutet, Stockholm, Sweden; 5 MSF, Berlin, Germany

**Keywords:** rifampicin-resistant TB, RR-TB, paediatric, household contact tracing, Central Asia

## Abstract

**BACKGROUND:**

Tajikistan has a high burden of rifampicin-resistant TB (RR-TB), with 2,700 new cases estimated for 2021 (28/100,000 population). TB is spread among household members through close interaction and children exposed through household contact progress to disease rapidly and frequently.

**METHODS:**

We retrospectively analysed programmatic data from household contact tracing in Dushanbe over 50 months. We calculated person-years of follow-up, contact tracing yield, number needed to screen (NNS) and number needed to test (NNT) to find one new case, and time to diagnosis.

**RESULTS:**

We screened 6,654 household contacts of 830 RR-TB index cases; 47 new RR-TB cases were detected, 43 in Year 1 and 4 in Years 2 or 3. Ten were aged <5 years; 46/47 had TB symptoms, 34/45 had chest radiographs consistent with TB, 11/35 were Xpert Ultra-positive, 29/32 were tuberculin skin test-positive and 28/47 had positive TB culture and phenotypic drug susceptibility results. The NNS to find one RR-TB case was 141.57 and the NNT was 34.49. The yields for different types of contacts were as follows: 0.7% for screened contacts, 2.9% for tested contacts, 17.0% for symptomatic contacts and 12.1% for symptomatic contacts aged below 5 years.

**CONCLUSION:**

RR-TB household contact tracing was feasible and productive in Tajikistan, a low middle-income country with an inefficient healthcare delivery system.

## Abstract (fr)

**CONTEXTE:**

Le Tadjikistan subit une forte prévalence de la TB résistante à la rifampicine (RR-TB), avec une estimation de 2 700 nouveaux cas pour l’année 2021 (28/100 000 habitants). La TB se propage entre les membres d’une même famille en raison d’interactions étroites entre les membres, et les enfants exposés dans ce contexte familial ont tendance à développer la maladie rapidement et fréquemment.

**MÉTHODES:**

Nous avons effectué une analyse rétrospective des données provenant du programme de recherche des contacts familiaux à Douchanbé sur une période de 50 mois. Nous avons calculé les années-personnes de suivi, le rendement de la recherche de contacts, déterminé le nombre nécessaire au dépistage (NNS) et le nombre nécessaire au test (NNT) pour identifier un nouveau cas, ainsi que le délai requis pour poser un diagnostic.

**RÉSULTATS:**

Nous avons dépisté 6 654 contacts familiaux de 830 cas index de RR-TB ; 47 nouveaux cas de RR-TB ont été détectés, 43 au cours de l’année 1 et 4 au cours de l’année 2 ou 3. Dix d’entre eux étaient âgés de moins de 5 ans ; 46/47 présentaient des symptômes de TB, 34/45 avaient des radiographies pulmonaires compatibles avec la TB, 11/35 étaient positifs au test Xpert Ultra, 29/32 étaient positifs au test cutané à la tuberculine et 28/47 avaient des résultats positifs à la culture de la TB ainsi qu’aux tests phénotypiques de susceptibilité aux médicaments. Le NNS pour trouver un cas de RR-TB était de 141,57, tandis que le NNT était de 34,49. Les rendements pour les différents types de contacts étaient les suivants : 0,7% pour les contacts dépistés, 2,9% pour les contacts testés, 17,0% pour les contacts symptomatiques et 12,1% pour les contacts symptomatiques âgés de moins de 5 ans.

**CONCLUSION:**

La recherche des contacts familiaux pour la RR-TB s’est révélée réalisable et fructueuse au Tadjikistan, un pays à revenu intermédiaire faible où le système de prestation des soins de santé présente des inefficacités.

TB contact tracing, especially among household contacts of TB index cases, has been shown to be an effective strategy for identifying those at highest risk of TB infection and active disease. This ensures that 1) diagnostics and treatment can be initiated early in active disease, 2) latent TB infection can be identified and treated with TB preventive therapy (TPT) in drug-susceptible TB (DS-TB) contacts, 3) vulnerable DS-TB contacts without evidence of infection (e.g., children aged <5 years and persons living with HIV (PLHIV) can be given TPT to prevent active disease, and 4) TPT can be considered for vulnerable drug-resistant TB (DR-TB) contacts and those with TB infection.^1^–^3^ The WHO has recently recommended that high-burden countries consider initiating preventive treatment for rifampicin-resistant TB (RR-TB) using levofloxacin-containing regimens.^3^ Active case-finding (ACF) involving whole communities has also been shown to reduce TB incidence – a goal relevant to the WHO End TB policy.^4^,^5^

Early detection and proper treatment of TB disease reduces transmission, morbidity and mortality. However, the most recent WHO World TB Report estimates that there are substantial proportions of undiagnosed, untreated pulmonary TB cases in communities.^6^ The proportion of active multidrug-resistant (MDR)/RR-TB cases among children who are diagnosed and treated is especially low, at 15%.^6^ Furthermore, children exposed to TB through household contact progress to active disease more rapidly and frequently.^7^ As detection of TB, especially RR-TB, among children remains challenging, symptom screening and sputum induction for RR-TB contacts may improve detection among this sub-group and prevent early morbidity and mortality.^7^

Although contact tracing of high-risk groups is widely included in global and national policies, including Tajikistan’s national TB guidelines, this activity is rarely prioritised outside of low-burden, resource-rich settings despite its benefits for reducing TB community transmission.^8^,^9^ TB incidence in Tajikistan is moderately high, with 8,600 incident cases estimated in 2021 (88/100,000 population), 4,301 of whom were missing from diagnosis and treatment.^10^ Approximately 270 incident TB cases in 2020 occurred among children. Tajikistan has a low rate of HIV/AIDS, with an estimated adult (15–49 years) prevalence of 0.2% in 2021.^9^,^11^ However, Tajikistan is recognised by the WHO as a country with a high burden of RR-TB.^12^ There were an estimated 2,700 incident RR-TB cases in 2021; however, only 2,167 cases were diagnosed and only 2,094 were started on treatment.^13^

The average household size in Tajikistan in 2021 was 5.56 persons;^14^ 21% of households had one child aged <16 years and an additional 55.4% had ≥2 children.^14^ Therefore, each RR-TB source case interacted with multiple other non-infected household members, especially children, likely resulting in additional people infected each year. A 2013 review of the Tajikistan National TB Programme noted no systematic approach by facility staff to evaluate the quality of contact tracing.^15^

Tajikistan guidelines dictate symptoms screening for all DS-TB and DR-TB household contacts, followed by diagnostic testing of contacts less than 5 years of age, contacts with HIV, and contacts aged ≥5 years with TB symptoms.^9^ Such households are to be visited within 2 weeks of identification and every 6 months for at least 2 years.

The Ministry of Health (MOH) has recently called for intensified efforts to scale up contact tracing and ACF through a systematic approach at the primary healthcare level, in polyclinics (general medical clinics), and involving general practitioners, paediatricians and community healthcare workers in rural areas.^9^

A 2016 review of DS-TB contact tracing in Dushanbe found successful treatment outcomes and TPT coverage. However, they observed that there is potential to enhance the scale of ACF and the implementation of national protocols by developing effective strategies, including the establishing of data linkages to enable routine monitoring and evaluation of the contact tracing process to ensure its quality and effectiveness.^16^

In this study, we sought to evaluate household contact tracing in Dushanbe, focusing on the yield, rate of progression following exposure and risk factors associated with a TB diagnosis.

## METHODS

We conducted a retrospective cohort study to analyse routinely collected programme data from Dushanbe, Tajikistan, and surrounding districts (population: 1,769,464) for 50 months (November 2017–December 2021). The study cohort included all household contacts of index patients, regardless of age, diagnosed with RR-TB during the study period. DS-TB contacts were excluded from the study. Household contact tracing data was linked to treatment registers and laboratory databases using a unique identifier.

### Objectives

The primary objective of our study was to describe the yield and efficiency of household contact tracing of RR-TB index cases performed according to Tajikistan national guidelines. In those referred for diagnostic testing, we wanted to know how many were subsequently diagnosed and treated for RR-TB in various subgroups. Our secondary objectives included the following parameters: 1) time to diagnosis, 2) person-years of follow-up, 3) the number needed to screen (NNS) to find a new RR-TB case, and 4) the number needed to test (NNT) to find a new RR-TB case.

### Definitions

Our study started in 2017 and all positive drug susceptibility testing (DST) results were recorded from patients enrolled prior to 2021; we therefore used pre-2021 WHO definitions of MDR-TB, RR-TB and extensively drug-resistant (XDR-TB).^17^

### Contact tracing procedure

We followed the Tajikistan national guideline for contact tracing and TB testing (Figure).^9^ Contact visits were done at the contact’s home within 14 days, and the household had the right to refuse visits. Follow-up visits took place every 6 months for 3 years. All household contacts (and/or their parents/guardians) were asked about TB signs/symptoms at each visit. Follow-up diagnostic testing was performed on contacts who were HIV-positive, contacts aged <5 years, and contacts aged >5 years with symptoms. TB sign/symptoms included current cough, poor weight gain or weight loss, fatigue, and night sweats, and reduced playfulness and activity in case of those aged <5 years. Diagnostic testing included chest radiography (CXR), tuberculin skin test (TST) or interferon-gamma release assay (IGRA), Xpert MTB/Rif Ultra (Cepheid, Sunnyvale, CA, USA) sputum testing, Mycobacteria Growth Indicator Tube (MGIT™; BD, Franklin Lakes, NJ, USA) culture and DST. DR-TB contacts (including children) with a positive TST or IGRA but without active disease were monitored but not routinely started on levofloxacin prophylaxis, although this strategy is a recent addition to the guidelines.^9^

**Figure:**
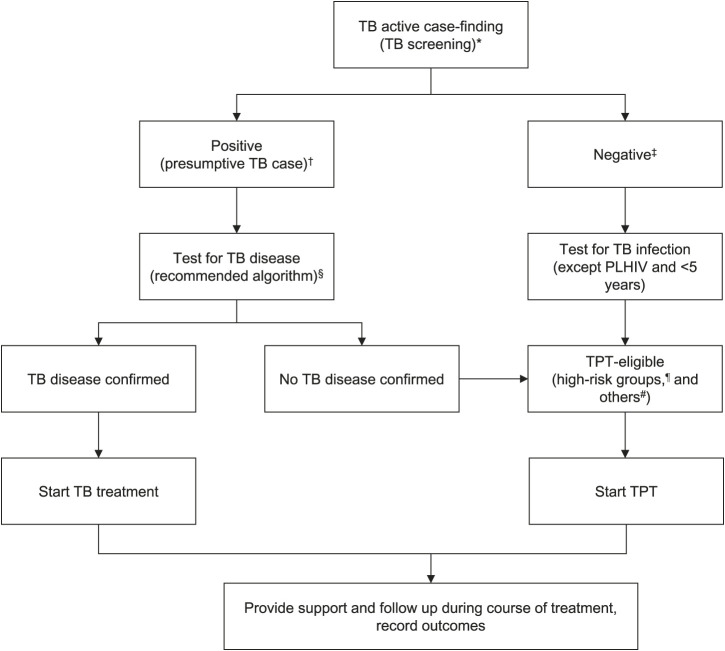
Approach to contact management. *Symptoms screen (current cough, poor weight gain or weight loss, fatigue, and night sweats, and reduced playfulness and activity in those <5 years). ^†^Patient has signs and/or symptoms suggestive of TB. ^‡^Patient has no TB signs/symptoms. ^§^Chest X-ray, TB skin test or interferon-gamma release assay, Xpert MTB/Rif Ultra sputum testing, MGIT culture and DST. ^¶^Children aged <5 years and PLHIV. ^#^Elderly, the immunocompromised, those with chronic comorbidities such as diabetes, those deemed high-risk by the TB Consilium. PLHIV = persons living with HIV; TPT = TB preventive therapy; MGIT™ = Mycobacteria Growth Indicator Tube.

### Statistical analysis

Our primary analysis describes the yield of household contact tracing overall (number of new cases identified in a group divided by all members of that group), by age, sex, presence or absence of TB symptoms, and in those with follow-up testing. In the secondary analysis, we determined the following: 1) person-years of follow-up: total days from earliest to last evaluation for all contacts who agreed to participate divided by 365; 2) the time from first assessment to disease progression: days from the first evaluation of those who agreed to participate to RR-TB confirmation; 3) the NNS: number of contacts screened divided by the number of new RR-TB cases identified; and 4) the NNT: number of contacts with follow-up testing divided by the number of new RR-TB cases identified.

### Eligibility criteria

All household contacts of RR-TB patients identified in participating MOH clinics in Dushanbe and surrounding districts who agreed to 6-monthly follow-up for 3 years were eligible.

### Exclusion criteria

Households that declined a visit and contacts whose record did not contain information on the index case or DST results were excluded.

### Ethical considerations

This study met the exemption criteria for ethical review as a retrospective review of routinely collected data of an activity as per the Tajikistan national TB guidelines.

## RESULTS

We visited 6,654 household contacts of 830 RR-TB index cases (average: 8.0 contacts/case) at least once from 2017 to 2021. The baseline characteristics of all contacts are shown in Table 1. We did not record the number or the baseline characteristics of contacts who declined to participate. We made one home visit for 4,036 (60.7%) contacts and more than one for 2,618 contacts (39.3%). Few contacts were visited for the full 36 months.

**Table 1:** Baseline characteristics of all contacts (*n* = 6,654)

	Male *n* (%)	Female *n* (%)	Total *n* (%)
<5 years	671 (10.1)	692 (10.4)	1,363 (20.5)
≥5 years	2,151 (32.3)	3,136 (47.1)	5,291* (79.5)
<5 years with TB signs and symptoms^†^	43 (0.6)	40 (0.6)	83 (1.2)
<5 years without TB signs and symptoms^‡^	628 (9.4)	652 (9.8)	1,280 (19.2)
≥5 years with TB signs and symptoms	85 (1.3)	101 (1.5)	186 (2.8)
≥5 years without TB signs and symptoms	2,068 (31.1)	3,037 (45.6)	5,105* (76.7)
<5 years referred for diagnostic testing^§^	671 (10.1)	692 (10.4)	1,363 (20.5)
≥5 years referred for diagnostic testing^¶^	85 (1.3)	101 (1.5)	186 (2.8)

*Four contacts of unknown gender aged ≥5 years with no TB signs or symptoms.

^†^Signs/symptoms include current cough, poor weight gain or weight loss, fatigue, night sweats, and reduced playfulness and activity in those <5 years of age.

^‡^TB signs/symptoms denied or not recorded.

^§^Regardless of symptoms, all <5-year contacts in Tajikistan are referred for diagnostic testing that includes TB skin testing or IGRA, chest X-ray, Xpert MTB/Rif Ultra testing and TB culture.

^¶^Contacts aged ≥5 years with TB signs/symptoms are referred for diagnostic testing in Tajikistan.

IGRA = interferon-gamma release assay.

We identified 47 new active RR-TB cases from 38 index cases, totalling over 2,908 person-years of follow-up (Table 2). The average follow-up was 159.6 days per individual and 405.6 days for contacts evaluated more than once. There were two additional contacts who were diagnosed and treated as RR-TB (1 MDR-TB, 1 XDR-TB) but whose records did not contain any information on the index case or DST results. These were excluded. We identified one new DS-TB patient and one new isoniazid-monoresistant patient.

**Table 2:** Characteristics of the 47 newly identified RR-TB cases among household contacts

	<5 years *n*	5–14 years *n*	>15 years *n*	Total *n/N* (%)
Male	6	13	3	22/47 (46.8)
Female	4	12	9	25/47 (53.2)
Diagnosed at first visit	6	12	6	24/47 (51.1)
Diagnosed in first year	9	23	11	43/47 (91.5)
TB signs/symptoms	10	24	12	46/47 (97.9)
CXR suggestive of TB	7	17	10	34/45 (75.6)
Positive TB skin test	8	17	4	29/32 (90.6)
Ultra RIF-resistant (total)	1	5	5	11/35 (31.4)
Xpert RIF-resistant and culture-negative, *n*	0	1	3	4
Positive TB culture	7	15	6	28/47 (59.6)
RR-TB, *n*	6	9	1	16
XDR-TB, *n*	1	6	5	12
RR-TB diagnosed clinically	2	10	3	15/47 (31.2)

RR-TB = RIF-resistant TB; CXR = chest X-ray; RIF = rifampicin; XDR-TB = extensively drug-resistant TB.

### New drug-resistant tuberculosis cases

Among 47 new RR-TB cases identified out of 6,654 contacts, the mean time from first assessment to disease progression and confirmation was 67 days (standard deviation: ±135). The average age of a new case was 11.8 years and over three-quarters were under 14 years of age. All but one patient had TB signs/symptoms. That patient was a 9 year-old female who was tested because of clinical suspicion and had a positive TB culture for XDR-TB. None of the 1,280 patients aged <5 years without TB signs/symptoms were confirmed with TB disease. Twenty-eight of the 47 new RR-TB cases had DST results: 16 were MDR-TB and 12 had XDR-TB. Seven of the 12 XDR-TB cases would be diagnosed as pre-XDR-TB according to the new classification system. Twenty-three patients with a positive TB culture were Ultra-negative. Of the 19 cases without a positive TB culture, four had a positive Ultra assay. The remaining 15 incident cases with a negative Ultra assay and negative TB culture were diagnosed on clinical and epidemiologic grounds by the Tajikistan TB Consilium of experts. Only two participants were known to be HIV-positive but neither were diagnosed with RR-TB. The HIV prevalence in Tajikistan is estimated to be 0.2%.^11^ We had DST information on 26 index cases of 33 new contact cases. In 10 cases (30.3%), the DST results were in concordance.

### Yields

The screening process yielded an overall rate of 0.7%. Among the contacts subjected to further testing, the yield was 2.9%, while those with TB symptoms had a yield of 17.0%. Specifically, contacts aged below 5 years with symptoms showed a yield of 12.1%, while those aged ≥5 years with symptoms had a yield of 19.4%. The NNS was 141.57 and the NNT was 34.49. Twenty-four cases were diagnosed at first assessment and 43 cases within the first year. All new RR-TB patients were treated (Table 3).

**Table 3:** Yield of contact tracing for incident RR-TB cases

	Male cases *n/N* (%)	Female cases *n/N* (%)	Overall *n/N* (%)
<5 years with TB signs/symptoms^*^	6/43 (14.0)	4/40 (10.0)	10/83 (12.0)
<5 years without signs/symptoms	0/628 (0.0)	0/652 (0.0)	0/1,280 (0.0)
≥5 years with TB signs/symptoms^†^	16/85 (18.8)	20/101 (19.8)	36/186 (19.4)
≥5 years without symptoms	0/2,068 (0.0)	1/3,037 (0.0)	1/5,105 (0.0)
Contacts with TB signs/symptoms^†^	22/129 (17.1)	24/141 (17.0)	46/270 (17.0)
All contacts^‡^	22/2,822 (0.8)	25/3,828 (0.7)	47/6,654 (0.7)
Contacts referred for diagnostic testing	22/788 (2.8)	25/833 (3.0)	47/1,621 (2.9)

*TB signs/symptoms include current cough, poor weight gain or weight loss, fatigue, night sweats, and reduced playfulness and activity in those <5 years of age.

^†^One female patient ≥5 years without TB signs/symptoms was referred for diagnostic testing because of clinical suspicion and had bacteriologically confirmed XDR-TB.

^‡^Gender unknown: 4.

RR-TB = rifampicin-resistant TB; XDR-TB = extensively drug-resistant TB.

## DISCUSSION

Key study yields were as follows: 12.1% in symptomatic <5-year-old household contacts of index patients with RR-TB and 0.0% in <5 years household contacts without signs/symptoms. Our study findings are consistent with the peer-reviewed literature, but ours is the first such report from Central Asia and we had more contacts per case (8.0) than most other studies.^2^,^18^–^26^

We reviewed four systematic review and meta-analysis publications.^7^,^18^–^20^ The methods and definitions used to trigger further investigation were highly context-dependent and had a large impact on results. We also reviewed eight stand-alone studies.^2^,^21^–^27^

Our overall yield was 0.7%, which is lower than those in the studies we reviewed, which had an average overall yield of 2.50 (range 0.96–6.50).^7^,^18^–^20^,^22^–^27^ DR-TB studies reported overall yields of 3.1%, 3.9% and 6.5%,^20^,^22^,^23^ and yields of 1.4% and 3.4% were reported in DR-TB subgroups within larger TB cohorts.^19^,^25^ The NNS is the reciprocal of the overall yield. Kranzer et al. found an overall yield of 2.2% and an NNS of 45 in HIV-positive contacts from low-income and middle-income countries with an HIV prevalence of >5% in TB cases.^18^ A recent article in the *New England Journal of Medicine* found an overall yield of 1.79% for ACF compared with 0.75% for passive case-finding.^2^ A cluster, randomised trial from Peru also found a higher yield with ACF vs. passive case-finding (incidence rate ratio of 1.51). This study also documented an incremental cost-effectiveness ratio of US$16,400 per disability-adjusted life-year averted for ACF.^21^ The authors estimated that 67 additional TB cases per 10,000 household contacts were screened annually with ACF. Several studies identified the greatest number of new cases in the first year.^2^,^19^,^27^

We found a yield of 12.1% for children aged <5 years with signs/symptoms. A study from Myanmar among household contacts of index patients with RR-TB reported a yield of 10.0% for children aged <5 years.^22^ Fox et al. found a similar yield of 10.0% among TB contacts aged <5 years, and Honjepari et al. reported a yield of 9.8% for this age group.^25^ These results underscore the importance of contact tracing in this high-risk age group for whom diagnosis is difficult.

We found a yield of 2.9% in contacts with further diagnostic testing, including 17.0% for all contacts with signs/symptoms and 19.4% for ≥5 years contacts with signs/symptoms. Comparisons to the literature are difficult, as most studies that we reviewed included some tests in their initial screening strategy and additional tests at the next step. However, Honjepari et al. reported a yield of 5.8% in all contacts with symptoms before further testing.^25^ Finally, a large study from Ethiopia found a yield of 15.1% in contacts who underwent further testing after a positive symptoms screen.^24^

The number of household members per index patient with RR-TB in our cohort was higher (8.0) than that reported in the last Tajikistan government survey in 2021 (average: 5.6) and higher than seven publications that we reviewed (average 4.3, range 2.0–6.0).^2^,^19^–^21^,^23^,^24^,^26^ A report from Papua New Guinea, however, documented an average of 8 household members of TB index patients vs. a national average of 5.7.^25^ TB is associated with poverty and overcrowding, and household size has been shown to be sensitive to socio-economic factors.^28^ We postulate that TB households in Tajikistan are poorer and larger than the average in Tajikistan and elsewhere.

There are several limitations to our study. We did not have the full results of diagnostic testing in all new RR-TB cases, and no information on total or negative HIV tests and the site of TB disease (pulmonary vs. extrapulmonary). We did not record information on contacts who declined to participate; however, our contact tracing teams agreed this number was low. Our teams estimated that two-thirds to three-quarters of families agreed to home visits for contact tracing and that visits were appreciated. The acceptability of this form of TB investigation has been previously documented at 91.1%.^25^ Our teams also felt that the most common reasons for declining a home visit and for the drop-off in follow-up visits were TB stigma and COVID-19 precautions. These reasons have been reported in the literature.^26^,^29^ Future research on this topic in our setting would provide good quality evidence and might also provide additional information on how to further increase acceptance.

Finally, we would like to highlight that the Ultra sputum assay missed several cases that had a positive TB culture and the TB culture missed a few Ultra-positive cases. Both tests should be performed in this vulnerable group.

## CONCLUSIONS

ACF through household contact tracing is important in settings such as Tajikistan as it serves to identify persons with RR-TB at an early stage, especially children less than 5 years of age, so that prompt and appropriate treatment can be initiated and advancing disease and complications can be limited. Our study also serves to validate WHO guidelines with respect to the key signs and symptoms in TB contacts and the most productive follow-up tests. RR-TB household contact tracing was an effective, feasible and productive intervention following national guidelines in Tajikistan, a low-income country with an inefficient healthcare delivery system. We believe that this intervention can and should be prioritised in similar settings.
